# Tolerance of Concurrent Adjuvant Radiation Therapy and Pembrolizumab for Triple Negative Breast Cancer: Real Life Experience

**DOI:** 10.1016/j.adro.2023.101384

**Published:** 2023-10-17

**Authors:** Thais Tison, Pierre Loap, Emilie Arnaud, Kim Cao, Solene Bringer, Manon Kissel, Safia Maaradji, Juliette Mainguene, Jean-Yves Pierga, Florence Lerebours, Anne Vincent-Salomon, Mariana Mirabelle, Francois-Clement Bidard, Delphine Loirat, Youlia M. Kirova

**Affiliations:** a Department of Radiation Oncology, Institut Curie, Paris, France; b Department of Radiation Oncology, Institut Curie, St Cloud, France; c Department of Medical Oncology, Institut Curie, Paris, France; d Department of Medical Oncology, Institut Curie, St Cloud, France; e Department of Diagnostic and Theranostic Medicine, Department of Pathology, Institut Curie, Paris, France; f Université Paris Sciences et Lettres, Paris, France; g Cardiology, Institut Mutualiste Montsouris, Paris, France; h Université de Versailles Saint-Quentin, Yvelines, France

## Abstract

**Purpose:**

The current standard-of-care management of locally advanced triple negative breast cancer (TNBC) is based on neoadjuvant chemo-immunotherapy with pembrolizumab, surgery, radiation therapy (RT), and adjuvant pembrolizumab. However, the safety of combining pembrolizumab with adjuvant breast RT has never been evaluated. This study evaluated the tolerance profile of concurrent pembrolizumab with adjuvant RT in patients with locally advanced TNBC.

**Methods and Materials:**

This bicentric ambispective study included all the patients with early and locally advanced TNBC who received neoadjuvant chemo-immunotherapy with pembrolizumab and adjuvant RT as part of their treatment. The tolerance profile of adjuvant RT was evaluated and compared in patients who received concurrent pembrolizumab and in patients for whom pembrolizumab was withheld.

**Results:**

Fifty-five patients were included between July 2021 and March 2023. Twenty-eight patients received adjuvant RT with concurrent pembrolizumab (RT+P group), and 27 patients had pembrolizumab withheld while receiving adjuvant RT (RT-only group). Two patients developed grade ≥3 toxicity (1 grade 3 pain in the RT+P group and 1 grade 3 radiodermatitis in the RT-only group), and there were no differences in terms of toxicity between the RT-only and the RT+P groups. No cardiac or pulmonary adverse event was reported during RT. With a median follow-up of 12 months (10-26), no patient relapsed.

**Conclusions:**

In this study of limited size, the authors did not find a difference between the RT-only and RT+P groups in terms of toxicity. More studies and longer follow-up may add to the strength of this evidence.

## Introduction

Triple negative breast cancer (TNBC) has the worst prognosis among all breast cancer subtypes.^1^ Antiprogrammed death 1 immune checkpoint inhibitors (ICI) have been found to reverse the protumoral immunomodulating microenvironment and increase the antitumor activity of tumor-infiltrating lymphocytes (TILs) in diverse cancer types.^2^^,^^3^ TNBC microenvironment is enriched in TILs,^4^^,^^5^ and ICI have consequently been evaluated in this indication with promising results^6^; in particular, for locally advanced TNBC, addition of pembrolizumab in the neoadjuvant and adjuvant setting has significantly improved the 3-year event-free survival from 76.8% to 84.5% in the phase 3 KEYNOTE-522 trial.^7^

Adjuvant breast radiation therapy (RT) improves local control and cancer-specific survival,^8^, ^9^, ^10^, ^11^ and continuing pembrolizumab during RT may theoretically improve tumor control in patients with TNBC. Additionally, RT may act synergistically with ICI by inducing an antitumoral microenvironment.^12^, ^13^, ^14^, ^15^, ^16^ However, the toxicity of combining RT with concurrent ICI has been a subject of concern,^17^ and the safety profile of combining adjuvant breast RT with pembrolizumab has not been specifically assessed in the KEYNOTE-522 trial. This study aimed to evaluate the tolerance profile of concurrent pembrolizumab with adjuvant RT in patients with locally advanced TNBC.

## Methods and Materials

### Population

This bicentric ambispective study was conducted in the department of radiation oncology [2 sites, situated in the Institut Curie (Paris and St Cloud)] and included all the patients with a locally advanced TNBC who received neoadjuvant chemo-immunotherapy with pembrolizumab and adjuvant RT as part of their treatment between July 2021 and March 2023. The patient registration was prospective for all patients receiving this treatment. This study was approved by the institutional review board of the Institut Curie. Our data collection followed our institutional review board's regulations of the Institut Curie Breast Cancer research and treatment study group with a waiver of informed consent, and the study was conducted according to the Declaration of Helsinki.

### Treatment

Neoadjuvant intravenous chemo-immunotherapy consisted of 4 cycles of pembrolizumab (200 mg, q3w), combined with 4 cycles of paclitaxel (80 mg/m² q1w) plus carboplatin (area under the curve 5, q3w or area under the curve 1.5, q1w), followed by 4 cycles of doxorubicin (60 mg/m² q3w) plus cyclophosphamide (600 mg/m² q3w). Patients underwent either breast conserving surgery or mastectomy, with sentinel lymph-node evaluation or axillary dissection. Patients were treated through the French Early Access Program for pembrolizumab. Neoadjuvant chemo-immunotherapy was administered to all patients.

Patients were scanned with both arms above the head using a Toshiba 64-slice computed tomography scan and images were transferred to the Aria treatment planning system (Varian Medical System) for treatment planning. The clinical target volumes, delineated according to the European Society for Radiotherapy and Oncology, included the whole breast with a tumor bed boost after breast conserving surgery or the chest wall after mastectomy.^18^ Regional lymph nodes (Berg's levels 2-4, interpectoral, internal mammary chain, with or without lower axillary lymph nodes) could be included based on the initial clinical presentation according to institutional guidelines. Planning treatment volumes were defined by an isotropic 5-mm margin around the clinical target volumes. Radiation therapy was delivered on linear accelerators. Patients were treated to breast (or chest wall) and lymph nodes were treated with volumetric-modulated arc therapy or intensity modulated RT with or without deep inspiration breath hold. The only 3-dimensional technique used in these series is the whole breast irradiation (without lymph nodes) in lateral position to avoid heart and lung irradiation.^19^

The fractionation scheme for chest wall irradiation with or without regional node irradiation (RNI) was 50 Gy in 25 fractions of 2 Gy. The fractionation scheme for breast RT was 40.05 Gy in 15 fractions of 2.67 Gy (with a 48-Gy simultaneous-integrated boost or a 16-Gy sequential additional boost) when no RNI was conducted or 50.4 Gy in 28 fractions of 1.8 Gy (with a 64.4 simultaneous-integrated boost) when RNI was performed.

Adjuvant pembrolizumab was administered at the dose of 200 mg q3w for up to 9 cycles or 400 mg q6w for a total dose of 1800 mg. Cardiac evaluation was done regularly in all patients. All patients were initially planned to receive concomitant pembrolizumab; however, pembrolizumab was suspended temporally or definitively due to pembrolizumab-related adverse events after evaluation by a multidisciplinary staff, including oncologists, cardiologists, pathologists, and radiologists. In case of clinical suspicion of immune-induced myocarditis, biopsies were also realized.

In these cases, other adjuvant systemic treatments were considered by the multidisciplinary meeting, such as capecitabine or olaparib.

### Statistical analyses

Patients were divided into 2 groups based on the administration of pembrolizumab during RT. All patients who received at least 1 dose of adjuvant pembrolizumab during RT were included in the concurrent pembrolizumab group (RT+P group), and patients who did not receive any adjuvant pembrolizumab cycle during RT (after a multidisciplinary decision to temporarily or definitively withhold it due to pembrolizumab-related adverse events) were included in the RT-only group (RT-only group). The primary endpoint was the tolerance profile of the RT, according to the treatment group and evaluated based on the Common Terminology Criteria for Adverse Events v5 scale. Secondary endpoints included the event-free survival and overall survival, from the date of diagnosis. Toxicity comparisons between the RT+P group and the RT-only group were performed using Fisher's exact tests, and posthoc power analyses with effect size calculation were additionally performed. Statistical significance was defined by a *P* value threshold of .05.

## Results

### Population

Between July 2021 and March 2023, 55 patients were included. The median follow-up since diagnosis was 12 (10-26) months.

Twenty-eight patients received adjuvant RT with concurrent pembrolizumab (RT+P group); 27 patients received adjuvant RT without concurrent pembrolizumab (RT-only group). Overall, no difference was observed between both groups in terms of patient demographics (Table 1), tumor characteristics (Table 1), surgery technique, or RT characteristics (Table 2).

**Table 1:** Patient and tumor characteristics

Variable		Whole population (n = 55)		Withheld pembrolizumab (n = 27)		Concurrent pembrolizumab (n = 28)		*P* value
Follow-up		12.00 (10.00-26.00)		12.00 (10.00-19.00)		13.00 (10.00-26.00)		.019
Age (median, range)		49.00 (29.00-69.00)		52.00 (32.00-69.00)		47.50 (29.00-64.00)		.058
BMI (median, range)		24.22 (16.80-39.54)		24.61 (16.80-39.54)		23.80 (17.69-36.51)		.805
WHO PS	0	53	96.36%	27	100%	26	92.86%	.488
	1	2	3.64%	0	0%	2	7.14%	
Mutation	No mutation	36	65.45%	17	62.96%	19	67.86%	.088
	*BRCA1*	8	14.55%	2	7.41%	6	21.43%	
	*BRCA2*	1	1.82%	0	0%	1	3.57%	
	*PALB2*	1	1.82%	1	3.7%	0	0%	
	Other	1	1.82%	0	0%	1	3.57%	
	NA	8	14.55%	7	25.93%	1	3.57%	
cT	T1	7	12.73%	4	14.81%	3	10.71%	.524
	T2	34	61.82%	16	59.26%	18	64.29%	
	T3	8	14.55%	3	11.11%	5	17.86%	
	T4a	1	1.82%	1	3.7%	0	0%	
	T4b	3	5.45%	1	3.7%	2	7.14%	
	T4c	0	0%	0	0%	0	0%	
	T4d	2	3.64%	2	7.41%	0	0%	
cN	N0	13	23.64%	7	25.93%	6	21.43%	.137
	N1	30	54.55%	11	40.74%	19	67.86%	
	N2	6	10.91%	4	14.81%	2	7.14%	
	N3	6	10.91%	5	18.52%	1	3.57%	
Ki67 (%, median, range)		70.00 (20.00-90.00)		60.00 (35.00-90.00)		70.00 (20.00-90.00)		.699
Grade	I	0	0%	0	0%	0	0%	1.000
	II	10	18.18%	5	18.52%	5	17.86%	
	III	45	81.82%	22	81.48%	23	82.14%	
Clinical stage	IIA	27	49.09%	14	51.85%	13	46.43%	.332
	IIB	14	25.45%	4	14.81%	10	35.71%	
	IIIA	5	9.09%	3	11.11%	2	7.14%	
	IIIB	4	7.27%	2	7.41%	2	7.14%	
	IIIC	5	9.09%	4	14.81%	1	3.57%	
TILs (%, median, range)		20.00 (0.00-95.00)		20.00 (0.00-95.00)		17.50 (0.00-95.00)		.845
Side	Right	28	50.91%	11	40.74%	17	60.71%	.237
	Left	26	47.27%	15	55.56%	11	39.29%	
	Bilateral	1	1.82%	1	3.7%	0	0%	

*Abbreviations:* BMI = body mass index; NA = not applicable; PS = performance status; TIL = tumor infiltrating lymphocytes; WHO = World Health Organization.

**Table 2:** Treatment characteristics

Variable		Whole population (n = 55)		Withheld pembrolizumab (n = 27)		Concurrent pembrolizumab (n = 28)		*P* value
Surgery	Tumorectomy (breast conserving surgery)	35	63.64%	18	66.67%	17	60.71%	.858
	Mastectomy	20	36.36 %	9	33.33%	11	39.29%	
Concurrent pembrolizumab	No	27	49.09%	27	100%	0	0%	NA
	Yes	28	50.91%	0	0%	28	100%	
RNI	No	24	43.64%	12	44.44%	12	42.86%	.746
	Yes (II-IV, IMC)	19	34.55%	10	37.04%	9	32.14%	
	Yes (I, II-IV, IMC)	3	5.45%	1	3.7%	2	7.14%	
	Yes (IV, IMC)	3	5.45%	2	7.41%	1	3.57%	
	Yes (NA)	4	7.27%	2	7.41%	2	7.14%	
	NA	2	3.64%	0	0%	2	7.14%	
Deep inspiration breath hold	No	33	60%	14	51.85%	19	67.86%	.437
	Yes	16	29.09%	9	33.33%	7	25%	
	NA	6	10.91 %	4	14.81%	2	7.14%	
Lateral isocentric decubitus	No	51	92.73%	26	96.3%	25	89.29%	.368
	Yes	2	3.64%	1	3.7%	1	3.57%	
	NA	2	3.64%	0	0%	2	7.14%	
Fractionation scheme	25 fractions (2 Gy)	15	27.27%	7	25.93%	8	28.57%	.826
	28 fractions (1.8 Gy) with SIB (2.3 Gy)	21	38.18%	10	37.04%	11	39.29%	
	15 fractions (2.67 Gy) and sequential boost (8 fractions)	10	18.18%	4	14.81%	6	21.43%	
	15 fractions (2.67 Gy) and sequential boost (5 fractions)	1	1.82%	1	3.7%	0	0%	
	25 fractions (2 Gy) and sequential boost (8 fractions)	2	3.64%	1	3.7%	1	3.57%	
	NA	6	10.91%	4	14.81%	2	7.14%	
Mean heart dose (Gy, median, range)		2.944 (0.157-5.852)		3.034 (0.157-5.852)		2.335 (0.443-4.800)		.566
Mean ipsilateral lung dose (Gy, median, range)		11.211 (1.300-15.000)		11.423 (1.300-14.100)		11.030 (1.314-15.000)		.95
V20 Gy ipsilateral lung (%, median, range)		19.4 (0.8-27.0)		21.00 (1.40-26.40)		18.15 (0.80-27.00)		.486
V30 Gy ipsilateral lung (%, median, range)		11.50 (0.30-16.10)		11.50 (0.90-16.10)		12.000 (0.300-15.700)		.521
Mean contralateral lung dose (Gy, median, range)		2.672 (0.008-5.407)		2.946 (0.008-5.407)		2.1455 (0.0370-4.2890)		.434
V20 Gy contralateral lung (%, median, range)		0.00000 (0.00000-0.90000)		0.0000 (0.0000-0.9000)		0.00000 (0.00000-0.60000)		.424
V30 Gy contralateral lung (%, median, range)		0.000000 (0.000000-0.100000)		0 (0-0)		0.000000 (0.000000-0.100000)		.332
Mean contralateral breast dose (Gy, median, range)		3.006 (0.224-5.293)		3.0240 (0.3040-5.2930)		3.001 (0.224-4.150)		.596
Mean thyroid dose (Gy, median, range)		6.884 (0.212-32.500)		7.152 (0.212-22.059)		6.884 (0.323-32.500)		.597
Radiotherapy suspension	No	50	90.91 %	23	85.19%	27	96.43%	.316
	Yes	4	7.27%	3	11.11%	1	3.57%	
	NA	1	1.82%	1	3.7%	0	0%	
Adjuvant pembrolizumab	No	19	34.55%	19	70.37%	0	0%	<.001
	Yes	35	63.64%	7	25.93%	28	100%	

*Abbreviations:* IMC = internal mammaey chain; NA = not applicable; RNI = regional nodal irradiation; SIB = simultaneous-integrated boost.

### Toxicity

Table 3 provides the maximal toxicities observed during the neoadjuvant chemo-immunotherapy. No difference was observed between the RT+P and RT-only groups, with the exception of myocarditis, which was only observed in the RT-only group (7 patients, 25%). Pembrolizumab-related myocarditis during neoadjuvant chemo-immunotherapy was an indication for definitive pembrolizumab suspension after evaluation by cardio-oncology staff.

**Table 3:** Neoadjuvant chemoimmunotherapy-related adverse events

Toxicity		CTCAE v5 grade	Whole population (n = 55)		Withheld pembrolizumab (n = 27)		Concomitant pembrolizumab (n = 28)		*P* value
Before radiotherapy	Myocarditis	2	1	1.82%	1	3.7%	0	0%	1.00
		3	7	12.73%	7	25.93%	0	0%	
		4	0	0%	0	0%	0	0%	
	Ventricular dysfunction	2	1	1.82%	1	3.7%	0	0%	1.00
		3	3	5.45%	3	11.11%	0	0%	
		4	0	0%	0	0%	0	0%	
	Cutaneous toxicity	1	2	3.64%	1	3.7%	1	3.57%	1.00
		2+	0	0%	0	0%	0	0%	
	Asthenia	1	26	47.27%	11	40.74%	15	53.57%	1.00
		2	1	1.82%	0	0%	1	3.57%	
		3+	0	0%	0	0%	0	0%	
	Dyspnea	1	2	3.64%	1	3.7%	1	3.57%	1.00
		2	3	5.45%	2	7.41%	1	3.57%	
		3+	0	0%	0	0%	0	0%	
	Cough	1	2	3.64%	1	3.7%	1	3.57%	1.00
		2+	0	0%	0	0%	0	0%	
	Hypothyroidy	1	0	0%	0	0%	0	0%	1.00
		2	6	10.91%	2	7.41%	4	14.29%	
		3+	0	0%	0	0 %	0	0%	
	Hyperthyroidy	1	1	1.82%	1	3.7%	0	0%	1.00
		2	1	1.82%	1	3.7%	0	0%	
		3	1	1.82%	1	3.7%	0	0%	
		4	0	0%	0	0%	0	0%	
	Adrenal insufficiency	1	1	1.82%	0	0%	1	3.57%	.33
		2	5	9.09%	4	14.81%	1	3.57%	
		3+	0	0%	0	0%	0	0%	
	Other toxicity	All grades	18	32.73%	12	44.44%	6	21.43%	

*Abbreviation:* CTCAE = Common Terminology Criteria for Adverse Events.

The maximal observed RT-related toxicities during RT and the subsequent follow-up are provided in Table 4. No patients experienced acute or late grade 4 to 5 adverse events. Two patients developed acute grade 3 toxicity: 1 grade 3 pain in the RT+P group (3.6%) and 1 grade 3 radiodermatitis (3.7%) in the RT-only group. No difference was observed in terms of grade 1 to 2 toxicity; in particular, no additional cardiac adverse event was reported.

**Table 4:** Adjuvant treatment-related adverse events

Toxicity	Toxicity	CTCAE v5 grade	Whole population (n = 55)		Withheld pembrolizumab (n = 27)		Concomitant pembrolizumab (n = 28)		*P* value	Power	w
After radiotherapy	Radiodermitis	1	37	67.27 %	19	70.37%	18	64.29%	.461	0.23	0.21
		2	9	16.36 %	3	11.11%	6	21.43%			
		3	1	1.82%	1	3.7%	0	0%			
		4	0	0%	0	0%	0	0%			
	Asthenia	1	22	40%	10	37.04%	12	42.86%	.614	0.12	0.15
		2	4	7.27%	1	3.7%	3	10.71%			
		3+	0	0%	0	0%	0	0%			
	Pain	1	10	18.18 %	7	25.93%	3	10.71%	.364	0.28	0.42
		2	0	0%	0	0%	0	0%			
		3	1	1.82%	0	0%	1	3.57%			
		4	0	0%	0	0%	0	0%			
	Myocarditis	1+	0	0%	0	0%	0	0%	NA	NA	NA
	Dyspnea	1	3	5.45%	0	0%	3	10.71%	1.000	0.28	0.42
		2	1	1.82%	0	0%	1	3.57%			
		3+	0	0%	0	0%	0	0%			
	Cough	1	4	7.27%	2	7.41%	2	7.14%	1.000	NA	NA
		2+	0	0%	0	0%	0	0%			
	Odynophagia	1	3	5.45%	2	7.41%	1	3.57%	1.000	0.07	0.17
		2	2	3.64%	1	3.7%	1	3.57%			
		3+	0	0%	0	0%	0	0%			
	Lymphoedema	1	5	9.09%	3	11.11%	2	7.14%	1.000	NA	NA
		2+	0	0%	0	0%	0	0%			

*Abbreviations:* CTCAE = Common Terminology Criteria for Adverse Events; NA = not applicable; w = effect size.

*P* values were calculated with Fisher's exact test. Posthoc power analyses were additionally performed.

### Survival

At data cut-off, no patients died or experienced local, regional, or metastatic relapse.

## Discussion

This ambispecific study demonstrated that pembrolizumab can be safely administered concomitantly with adjuvant RT in patients with locally advanced TNBC. This combination allows maintaining a systemic treatment in these high-risk patients while performing adjuvant RT, which can last from 3 to 5 weeks. In addition, it should be underlined that most treatment-related adverse events occurred during the neoadjuvant phase in the KEYNOTE-522 trial.^7^ In fact, in this series, the main cause of discontinuation of pembrolizumab was related to suspicion of cardiac adverse events occurring during the neoadjuvant treatment and was related to the injection of pembrolizumab or the chemotherapy. We observed 12.72% myocarditis based on the international cardio-oncology criteria; none were steroid refractory or resulted in death. These real life data are interesting because they show that all myocarditis happened before RT and did not affect the administration of RT. In this context, the optimal RT technique and cardiac dose constraints after ICI-induced myocarditis are unknown, and late cardiotoxicity studies will be needed to precisely determine these points. Cardiac sparing techniques, such as deep inspiration breath hold, proton therapy, or alternative positioning (such as isocentric lateral decubitus) could be considered.^19^

The rate of observed toxicities in patients treated with immuno-RT varies significantly between studies for a number of reasons, such as tumor localization, patient characteristics, and delivered treatment.^13^^,^^20^ The KEYNOTE-001 randomized controlled trial^21^ evaluating pembrolizumab in metastatic non-small cell lung cancers found that patients who had previously undergone thoracic radiation were more likely to experience pulmonary adverse events than patients who had never been irradiated (63% vs 40%). However, current available data on brain, liver, lung, and prostate cancers do not support a significant risk increase of treatment-related toxicity when combining immunotherapy with RT.^20^^,^^22^

In TNBC, available data on radio-immunotherapy are scarce and are limited to the metastatic setting. A phase 2 study evaluating the safety of concomitant administration of pembrolizumab with palliative RT in metastatic patients showed that the association was well tolerated, mainly with low-grade, skin-related adverse events related to RT.^23^

On the other hand, as we previously mentioned, when planning adjuvant breast RT for patients with early TNBC with underlying immuno-induced myocarditis, we suggest that unintentional low-dose exposure to the heart should be taken into account to limit the theoretical risk of auto-immune response reactivation against the myocardium, while simultaneously respecting validated maximal dose constraints to cardiac substructures. Such considerations may lead to the selection of specific alternative RT techniques.^24^

The ambispecific and bicentric nature of this study, as well as its limited size, remain the main limitations, which do not allow us to draw definitive conclusions on the toxicity equivalence or the efficacy of combining adjuvant RT with pembrolizumab in this patient population. However, our results suggest that this approach is safe and could be considered in future phase 2 to 3 trials in patients with TNBC. Nevertheless, a longer-term follow-up will be required to evaluate the risk of late lung and cardiac adverse events.

## Conclusion

In this study of limited size, it was not found a difference between the RT-only and RT+P groups in terms of toxicity. More studies and longer follow-up may add to the strength of this evidence.

## Disclosures

The authors declare that they have no competing interests.
